# Neglected Intrauterine Device Migration Complications: Case Reports

**DOI:** 10.1089/whr.2022.0099

**Published:** 2023-01-18

**Authors:** Audrey S. Koh

**Affiliations:** Department of Obstetrics, Gynecology and Reproductive Sciences, School of Medicine, University of California, San Francisco, San Francisco, California, USA.

**Keywords:** intrauterine device, migration, perforation, IUD complications, annual examination, case report

## Abstract

This series of four cases describes unusual intrauterine device (IUD) migration complications, associated etiologic factors, and their management. Recent increases in sociopolitical and medical forces worldwide contributed to inattention to these patients with subclinical IUD complications. The international movement of patients, delays in gynecologic care due to the COVID-19 pandemic, lack of health care access, shifts away from annual examinations, and patients' lack of medical records or IUD awareness can lead to long durations of neglected IUDs and associated complications. These complications might have been avoided or minimized with regular gynecologic health care. Physicians should consider moving toward routine examinations of all IUD patients, rather than away from annual examinations as has been promulgated by some medical societies. Patient education is needed on the importance of routine monitoring of IUDs and retention of device records. Clinicians should become familiar with IUDs from around the world as well as devices no longer being prescribed, as their appearance, associated complications, and presentations may differ from locally currently available devices.

## Introduction

Intrauterine device (IUD) usage has increased to an estimated 159 million worldwide users in 2019, constituting 8.4% of women of reproductive age.^1^ With rising usage has come an increase in IUD complications. This series of four case reports showcases unusual and disparate complications of IUD migration, all likely exacerbated by prolonged neglect: (1) A Lippes Loop, forgotten for three to five decades, presenting as a vesicular stone; (2) a Gynefix IUD with retracted strings, *in vivo* for more than two decades in a patient who did not even know she had an IUD; (3) a partially embedded Paraguard in a postmenopausal patient; and (4) a Kyleena with strings protruding ectopically from the cervix, distant from the cervical os.

These case reports highlight medical and social factors contributing to sparse medical care in patients whose IUD complications were thus undiagnosed and allowed to worsen. The recognition that these complications might have been avoided or mitigated by routine surveillance bolsters consideration for regular checkups for any patient with an IUD, in contrast to the position of several medical societies against yearly gynecologic examinations.

## Background

Worldwide, IUDs are an increasingly popular, effective, and generally trouble-free contraceptive choice. IUD prevalence is particularly high among reproductive age women in Korea (46.9%), Uzbekistan (36.9%), and Turkmenistan (30.8%). Prevalence in Vietnam, China, and Cuba is 27.0%, 26.2%, and 23.3%, respectively, whereas in the United States and Europe prevalence figures are 8.3 and 8.1%, respectively.^1^ China has long been the nation with the largest number of IUD users, an estimated 88.5 million women in 2019.^2,3^

Although there are several types of IUD complications, this article focuses on device migration or malposition. Consequences of IUD migration are nonvisualized strings; IUD expulsion; unintended—including increased risk of ectopic—pregnancy; displacement within the uterine cavity or embedment in the myometrium; IUD fracture; and transuterine perforation into the peritoneal cavity and/or extrauterine tissue or organs.

Several recent medical, social, and political factors contribute to the likelihood that clinicians will encounter unfamiliar IUDs OR IUD complications that have festered over time. Increasing geographic mobility of patients worldwide brings patients with devices novel to their new countries of residence. Some patients have limited knowledge of or access to their IUD records and this can be worsened—rather than improved—with the proliferation of uncoordinated electronic medical records.

The COVID-19 pandemic and political, wartime, economic, and climate-change refugees have added to the movement of patients domestically and internationally, resulting in many patients delaying their usual health care, and long intervals between IUD checks. In the United States, the percentage of women aged 21–29 years who received a pelvic examination in the past 12 months decreased from 69.1% in 1995 to 56.5% during 2015–2017, the last years for which statistics are available. There were lower pelvic examination rates associated with Hispanic race (45.4%), lower education level (51.5% if no high school graduation), and lack of medical insurance coverage (38.9% of uninsured).^4^

The very success of IUDs as long-acting contraceptives plus their efficacy as mollifiers of molimina may also be associated with sparser gynecologic appointments. Finally, patient misunderstanding that longer Papanicolaou (Pap) smear intervals means that regular healthcare is unnecessary, plus conflicting guidelines regarding annual preventive health care for young women^5–8^ have further contributed to longer appointment intervals. These factors may lead to an increased occurrence of complications in expired or older IUDs, undiscovered IUD complications, and possible worsening of complication severity.

### Presentation of case 1

**FIG. 1.**
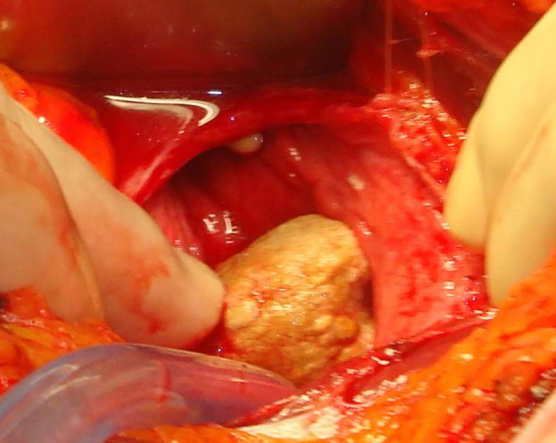
Large calculus perforating bladder wall.

A 75-year-old nulligravid woman presented to her primary care physician with microhematuria. She was referred to a urologist who found a 3.5-cm calculus and planned removal *via* cystotomy. Intraoperatively, the urologist called for this gynecologist's aide when the calculus was found to perforate the lateral bladder wall with a foreign body therein (Fig. 1).

**FIG. 2.**
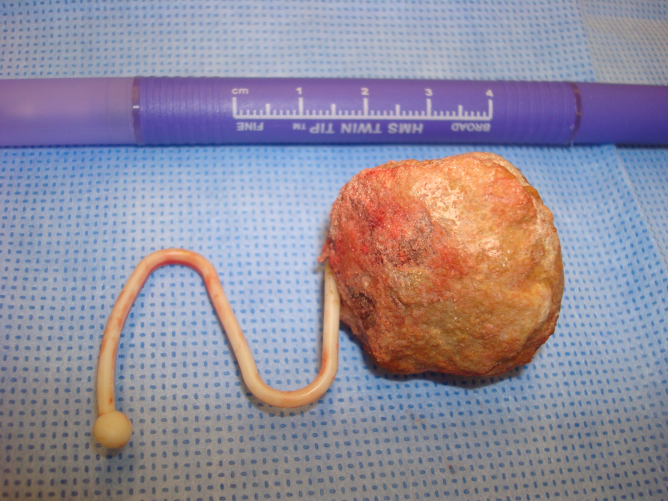
3.5-cm bladder calculus with protruding IUD. IUD, intrauterine device.

**FIG. 3.**
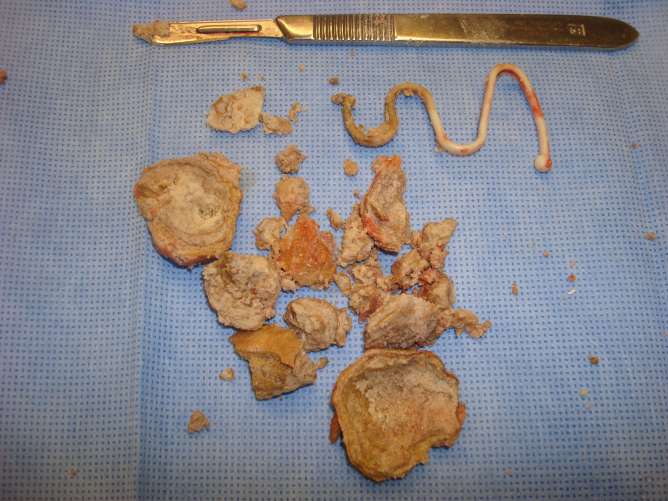
Lippes Loop IUD with fragmented bladder calculus.

The entire calculus with protruding IUD was gently brought through the perforation and the cystotomy repaired. There were no fistula or adhesions and the uterine serosa was intact. The calculus was incised and a Lippes Loop identified (Figs. 2 and 3). After surgery the patient was queried and had no recollection whatsoever of IUD placement. She could not state who or where her gynecologist was before the 1980s. Her hematuria resolved.

### Discussion of case 1

This case illustrates an *in vivo* Lippes Loop of extremely lengthy uncertain duration, due to lack of patient records or recollection, and due to mild to no symptomatology. The Lippes Loop was first distributed in the United States in 1962, becoming the most popularly used IUD in the United States soon thereafter. It was in worldwide usage until the early 1980s^9^ and removed from the market in 1986. Considering this timeline, this patient could have had her IUD for a period of 34 to, potentially, 58 years. The longest reported case of any retained IUD was a Lippes Loop, *in situ* for 50 years.^10^ It was removed upon discovery from the 74-year-old patient, who had been symptom free.

Transuterine perforation of an IUD is rare. In a multinational prospective study of >61,000 women, perforation occurred in ∼1 of 1000 insertions, with no difference between the two types studied, levonorgestrel and copper IUDs.^11^ Once transuterine perforation has occurred, the bladder is a common migration site, sometimes with calculus formation^12^; it was reported in 23 of 165 cases of extrauterine displacement.^13^ Removal of the IUD and intravesical stone has been managed with cystoscopy and mechanical removal or lithotripsy, cystotomy particularly when bladder penetration is incomplete, and/or laparoscopy.^14^

This case is a reminder that IUDs no longer in distribution can be harbored in patients and present with unexpected complications, particularly when the patient recalls no history of even having an IUD.

### Presentation of case 2

A 52-year-old postmenopausal patient, gravida 1 para 1, recent immigrant to the United States, presented for preventive gynecologic care. She reported regular menses until 2 years prior. While obtaining a Pap smear, a string was barely seen high in the canal. Upon questioning, the patient denied having an IUD, with no recollection of insertion or IUD discussions during her erratic prior medical care. She had had a vaginal delivery 26 years previously, in China.

**FIG. 4.**
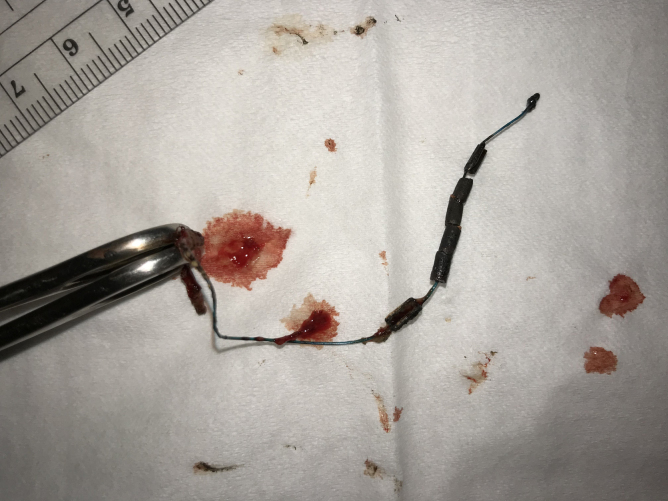
Encrusted GyneFix 330 copper IUD.

With the likelihood that this foreign body was an unnecessary IUD, removal was embarked upon. It could not be grasped by the string. Thus, topical anesthetic spray was applied, the cervix dilated and a uterine hook used for removal. It was encrusted but appeared intact, although it was an IUD unfamiliar to this gynecologist (Fig. 4). It was identified as a GyneFix 330 copper frameless IUD. Owing to the device's long residence *in situ* and to confirm its complete removal, office ultrasound was done demonstrating an empty cavity, no echogenic fragments, and a thin regular endometrium of 3 mm thickness.

### Discussion of case 2

This case illustrates the removal of an IUD with a retracted string requiring intrauterine instrumentation. The GyneFix IUD has been used mainly in the European Union countries, Turkey, China, and Vietnam. It was developed in Belgium and has been manufactured in China for distribution in Asia. The proximal end of the device has a knotted thread that anchors into the fundal myometrium, securing the device for decades of usage. This IUD had likely been in place for 26 years. Owing to patient and physician unfamiliarity with this device, a transvaginal ultrasound was done to rule out complications and to verify the removal of the entire intact device.

A Canadian review compared 10 years of IUD removals from patients whose devices had been inserted in China (327 patients) versus inserted in Canada (154 patients). It showed that successful in-office removals were accomplished in 72.3% versus 95.8% of China- and Canada-origin IUDs, respectively. This was attributed to the stark differences in accessibility of an IUD string, visible in only 13.4% versus 93.7% of China- and Canada-origin IUDs, respectively. The GyneFix was uncommonly found in 3.2% of IUDs of China origin.^15^

Historically, there has been predominant usage of a wide variety of stringless IUDs in China.^16^ In the Canadian study, 86.6% of IUDs inserted in China had inaccessible or no strings whatsoever.^15^ In service of the 1980 “one-child” policy in the People's Republic of China, there were many involuntary IUD insertions,^17,18^ and it has been suggested that IUDs were designed or adjusted to be more difficult to extract, with no or very shortened strings.^16,18^

Interestingly, an article in the popular press described a patient subjected to laparoscopy to search for a presumed fractured IUD, after her physician removed an IUD (a GyneFix) without arms.^19^ This highlights the importance of researching the appearance of IUDs from around the globe and from prior decades when formulating care of the IUD-bearing patient or encountering a novel device.^20–22^

### Presentation of case 3

A 59-year-old patient, postmenopausal for 2 years, presented for a routine gynecologic examination. She requested IUD removal. She could not remember what type of device she had, nor when it was placed, but estimated that it had been 12 years prior. She recounted that her prior gynecologic examination had been 4 years prior, when she was menstruating regularly. Documentation of her device insertion was not available due to nonintegrated electronic health records.

**FIG. 5.**
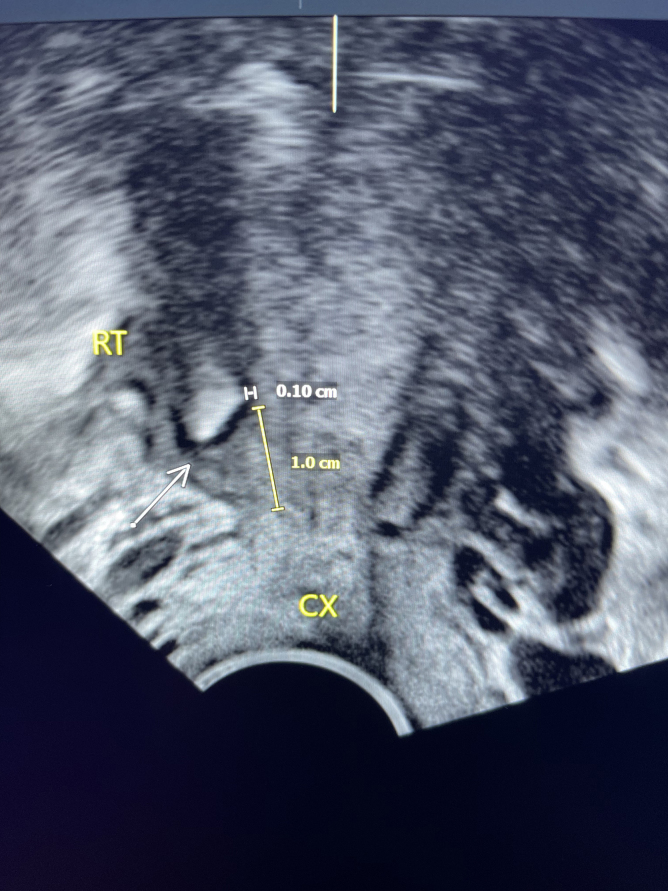
Three-dimensional ultrasound image: arrow points to IUD fragment, 0.10 cm below the endometrial surface and 1.0 cm from the internal os (“CX”), localized to the right myometrium (“RT”).

**FIG. 6.**
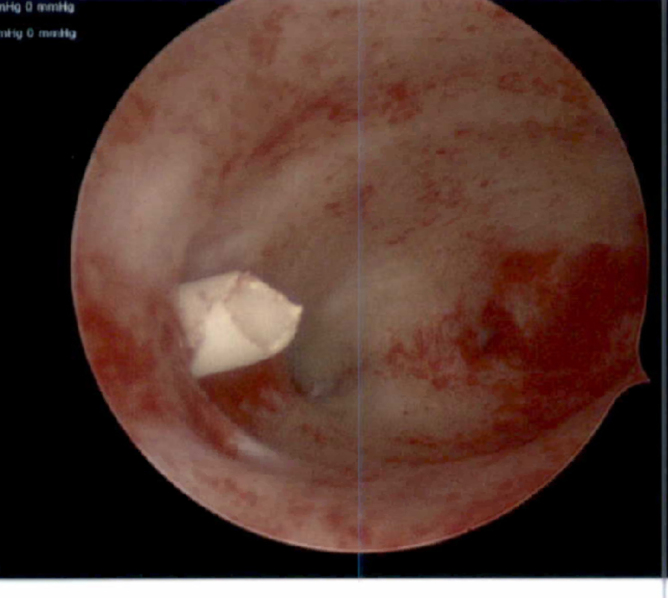
Hysteroscopic image: shorn IUD fragment visible at right lateral myometrium.

**FIG. 7.**
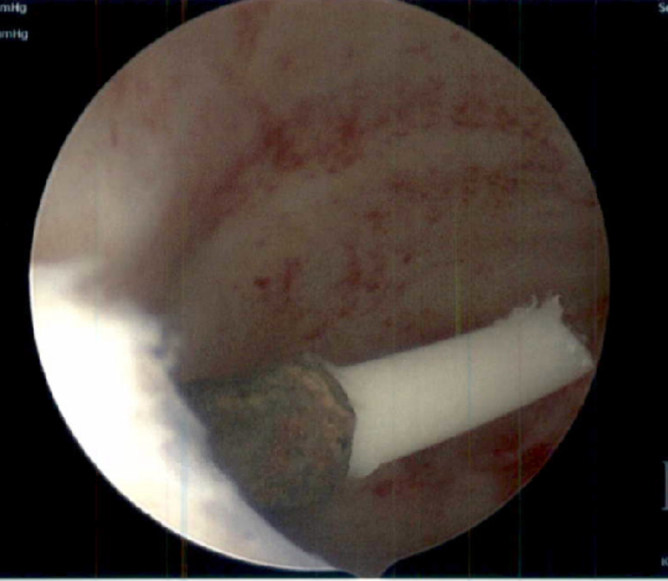
Hysteroscopic image: IUD arm partially pulled out of myometrium. Corroded copper coils visible.

**FIG. 8.**
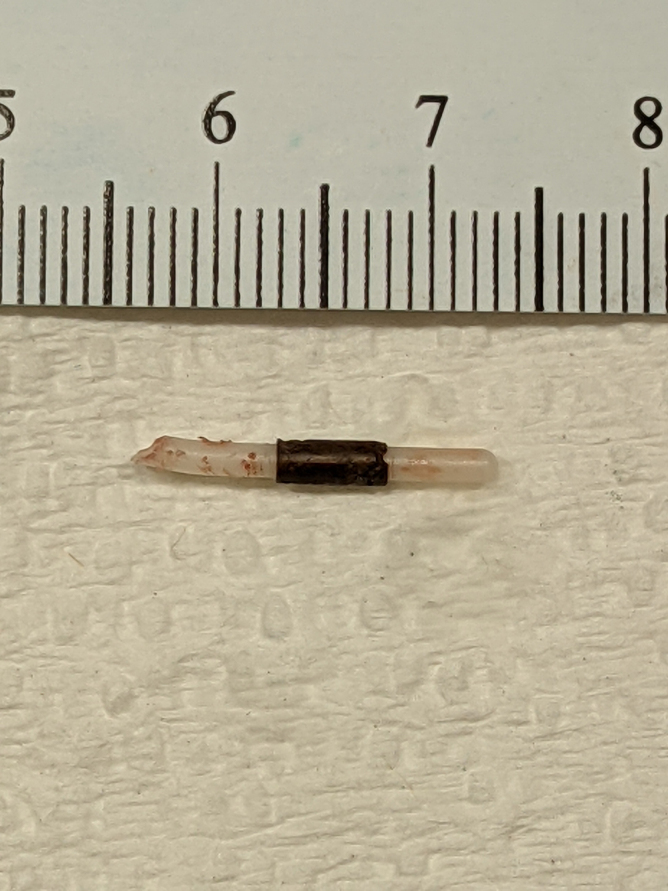
Fractured Copper T380A arm, 1.6 cm length with coil corrosion.

The strings were grasped with traction of customary smoothness and force. A partial Copper T380A (Paraguard) IUD came out, with one arm missing. The cervix was dilated and the cavity explored in an effort to extract the missing arm, but this was unsuccessful. Transvaginal three-dimensional ultrasound showed the arm embedded in the myometrium, but below the endometrial mucosal surface (Fig. 5). The embedded fragment was 16 mm long. Using hysteroscopy, the fragment was detected and extracted with a grasper under direct vision (Figs. 6–8).

### Discussion of case 3

In a retrospective case series, IUD fractures were reported at a higher rate with copper, as opposed to levonorgestrel, IUDs.^23^ Studies of European copper IUDs showed mechanical fatigue and copper wire dissolution with increased time *in utero.* These factors can increase the risk of copper IUD fracture with increased duration of use.^24,25^ This IUD had already migrated when removal was attempted; the partial embedment plus possible mechanical fatigue contributed to the IUD's fracture when traction was applied.

There is mixed advice on treatment of a device embedded in the myometrium or completely migrated into the peritoneal cavity when the patient is asymptomatic, but the World Health Organization recommends removal.^26,27^ Copper IUDs are suspected to incite a greater inflammatory reaction and adhesive disease compared with levonorgestrel IUDs,^28^ and some studies found more severe adhesions with increased duration in the peritoneal cavity,^29^ so there is stronger opinion supporting the retrieval of copper IUDs.

This case of a fractured IUD was marked by a lack of medical records and IUD monitoring. The patient had missed or rescheduled several annual examinations for her convenience, and then due to COVID-19 pandemic concerns. During those 4 years she passed into menopause. Uterine atrophy from hypoestrogenism (*e.g.*, postpartum, with lactation, in menopause) is theorized to contribute to IUD migration and myometrial embedment.^27,29,30^ The unmonitored years during her menopausal transition may have contributed to her IUD corroding and becoming more firmly embedded after initial migration from hypoestrogenic uterine changes, thus contributing to its fracture.

### Presentation of case 4

**FIG. 9.**
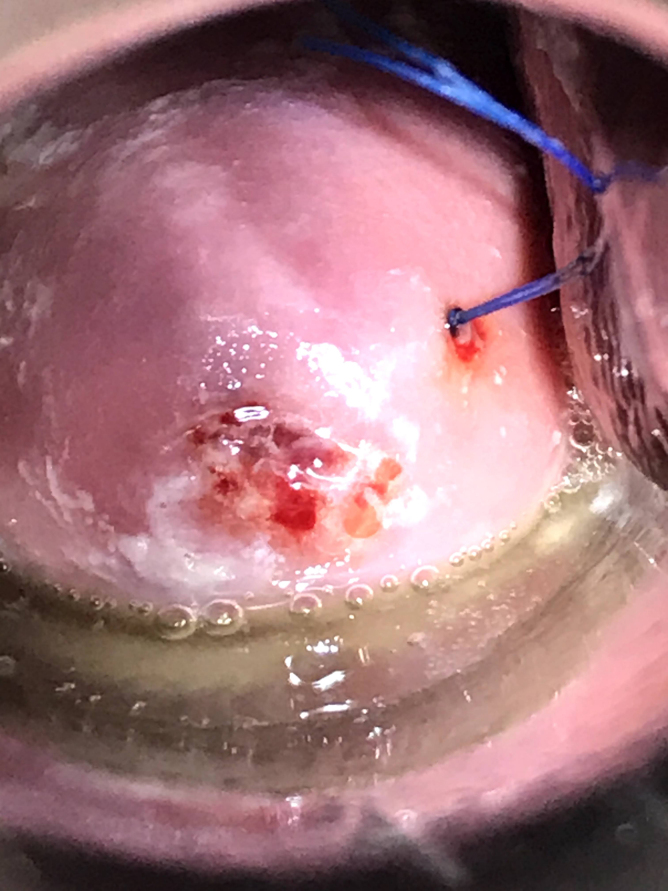
IUD strings egressing punctum, separate, and peripheral from the normal cervical os.

**FIG. 10.**
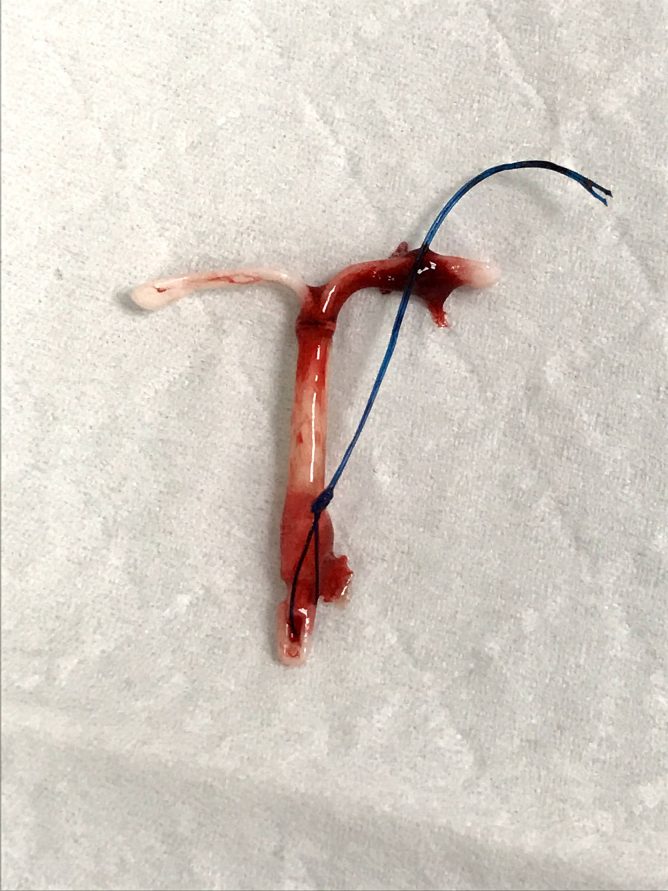
Intact Kyleena IUD, removed.

A 28-year-old nulligravida presented for removal of an expiring, and insertion of a new IUD. She was last seen in the office 2 years prior with IUD strings noted normally at the cervix. This time, the strings were found protruding through the cervical stroma *via* a small punctum, 1 cm-peripheral to her cervical os (Fig. 9). A paracervical block was administered and her IUD removed intact with a hook device. The IUD strings retracted into the uterus and then out the cervical os, along with her intact Kyleena (Fig. 10).

### Discussion of case 4

This case of IUD strings migrating through a new aperture in the cervix has never been previously reported. The Kyleena insertion had gone smoothly and the patient had last been checked 2 years previously. The patient had stable oligomenorrhea after her IUD insertion, so presumably had a continuous pharmacological effect. The patient was a nulligravida with no other risk factors for device migration, nor had she had gynecologic procedures or uterine manipulations that might have caused IUD misplacement. It is hard to conceive of how the strings could have retracted up into the cervical canal and then back out through a neo-aperture.

Had she been unmonitored for an even longer duration, her IUD could have become deeply embedded or further displaced.

## Summary

These four disparate cases of IUD complications illustrate subclinical problems that were very likely worsened by prolonged medical neglect. For IUD patients, a lack of bothersome symptoms does not necessarily equate with a benign clinical course. In these times of increased geographic movement of patients sometimes without access to their medical records, changing nonintegrated electronic medical record systems, lengthening intervals between gynecologic appointments, and the panoply of IUDs used historically and around the world, it is important that patients know the details of their IUDs. It should be reinforced that patients retain documentation of their IUD type, lot number, insertion, and expiration dates. Patients should be counseled on the importance of monitoring IUD positioning, especially in case of suspicious symptoms such as pain or unscheduled bleeding.

## Conclusion

This case series of IUD complications compounded by inattention leads to these takeaway recommendations: (1) Annual monitoring of *all* patients with IUDs that aligns with the American College of Obstetrics and Gynecology guideline,^8^ despite a lack of consensus for routine well-woman examinations among other medical organizations.^5–7^ (2) Patient education and counseling on the importance of retaining their IUD-specific information and the primacy of regular monitoring. (3) A low threshold for ultrasonographic or radiological confirmation of IUD positioning, since embedment and/or perforation have been demonstrated even when strings are still visible at the cervix. (4) That gynecologists, radiologists, urologists, general surgeons, and primary care providers familiarize themselves with the variety of IUDs used worldwide—both currently and historically—as many outdated IUDs remain *in vivo* and global travel and patient migration are increasing. In conclusion, “benign neglect” is a fallacy for IUD patients.

## Abbreviations Used

IUD: intrauterine device
Pap: Papanicolaou
